# Adaptive Spontaneous Transitions between Two Mechanisms of Numerical Averaging

**DOI:** 10.1038/srep10415

**Published:** 2015-06-04

**Authors:** Noam Brezis, Zohar Z. Bronfman, Marius Usher

**Affiliations:** 1 School of Psychology, Tel-Aviv University; 2 The Cohn Institute for the History and Philosophy of Science and Ideas, Tel-Aviv University; 3 Sagol School of Neuroscience, Tel-Aviv University

## Abstract

We investigated the mechanism with which humans estimate numerical averages. Participants were presented with 4, 8 or 16 (two-digit) numbers, serially and rapidly (2 numerals/second) and were instructed to convey the sequence average. As predicted by a dual, but not a single-component account, we found a non-monotonic influence of set-size on accuracy. Moreover, we observed a marked decrease in RT as set-size increases and RT-accuracy tradeoff in the 4-, but not in the 16-number condition. These results indicate that in accordance with the normative directive, participants spontaneously employ analytic/sequential thinking in the 4-number condition and intuitive/holistic thinking in the 16-number condition. When the presentation rate is extreme (10 items/sec) we find that, while performance still remains high, the estimations are now based on intuitive processing. The results are accounted for by a computational model postulating population-coding underlying intuitive-averaging and working-memory-mediated symbolic procedures underlying analytical-averaging, with flexible allocation between the two.

Averaging numerical information is essential in the formation of preferences about a variety of items, from stocks and grocery lists to participants in a contest, as well as for making decisions between alternatives characterized by numerical values^1^,^2^,^3^,^4^. While previous work has indicated that human observers can generate quite accurate estimations of numerical values^5^,^6^, they had mostly relied on group estimations. Here we set out to investigate the ability of participants to carry out such estimations and the underlying mechanisms, using a psychophysical, within participant approach.

In particular, we are interested in distinguishing between two potential estimation mechanisms: a) an analytic one (also termed the exact system) that is based on rule-governed serial operations, performed on values held in working-memory; and b) an intuitive one (also termed the approximate system) that is based on parallel processes operating on analog and fuzzy representations (7,8,9,10,11; but see^12^,^13^). According to this schema, numerical intuition is considered to reflect reliance on perceptual-like mechanisms, such as those that operate in statistical estimations of the numerosity or size of visual elements^14^,^15^,^16^, while analytic calculations are seen as a product of a symbolic pathway used for the sequential application of arithmetic operations or heuristics^17^,^18^,^19^. These two mechanisms differ in their functional properties: the intuitive system is automatic, rapid and high-in-capacity, yet capable only of an approximate (coarse) estimate at the single-item level, while conversely, the analytic/symbolic system is precise, but mediated by working memory and thus restricted in capacity. Importantly, these functional discrepancies render each system optimal under different task contingencies.

Consider, for example, a situation in which one must assess the average-value (e.g., prices, quality-evaluations) of a certain quantity of items that are only briefly presented without being able to take notes. When presented with only a few items (i.e., within working memory capacity), and assuming no time pressure, one should better apply an analytic solution, which involves a sequential application of numerical operations. Nonetheless, as the sequence-length increases, a growing amount of information is excluded from the already occupied working memory, resulting in a deteriorated reliability of the analytic solution. On the other hand, since the intuitive system has a higher capacity than the analytic one^20^,^21^,^22^, any additional information should theoretically improve its accuracy, since uncorrelated noise at the single-item level averages-out (see Results). Therefore, from a normative point of view, when the amount of information reaches a certain threshold, or when the information is presented at a speed that exceeds the temporal capability of the serial analytical system^23^, one should shift from analytic thinking to intuition.

To investigate these issues in a controlled environment, we have conducted four experiments in which participants were presented with sequences of two-digit numbers and required to produce an estimation of the average. The first two experiments (Exp. 1, N = 18, skewed distributions; Exp. 2, N = 18, normal distributions) used a moderate presentation rate of 500 ms per numeral, and no RT pressure allowing both strategies to operate, in order to probe for spontaneous strategy changes with set-size. In the last two experiments we probed reliance on intuition alone, by presenting the numbers at an extremely rapid rate (Exp. 3, N = 18, 100 ms per numeral), or by explicitly limiting the response time (RT) (Exp. 4; N = 18, RT limit of 2.5 sec; see Method section for full description; and Fig. 1 for an illustration of a typical trial).

Our aims are thus two-fold: (i) to demonstrate that individuals are able to discriminate between the averages of rapidly-presented number sequences, and test whether participants spontaneously adapt their strategy as a function of set-size to enhance performance; (ii) to offer a dual-component computational model that accounts for these abilities.

## Results

We first present the qualitative predictions of the two components of the computational model that will allow us to characterize the behavioral signatures of each of the systems, separately.

### Computational model: predictions for the intuitive and analytic systems

The model’s *intuitive-component* is grounded on neurophysiological evidence, demonstrating that approximate numerosity and number representations are coded in the parietal cortex of primates^24^,^25^ and humans^26^,^27^. The model follows Dehaene and colleagues^28^,^29^,^30^,^31^ to assume that symbolic numbers activate broad numerosity tuned neural detectors. We assume that when presented with a sequence of numerosity displays or numbers, the sequence’s average can be estimated from the number-tuned neural activation profile, by weighting the contribution of each neuron’s activity according to its preferred number/quantity [i.e., extracting a population vector;^32^; see *Computational Model Section* for additional description]. As we show in the simulations below, this model predicts that estimations of a sequence’s average improve with increasing number of samples (Fig. 2 Lower Panel, right-hand; blue line), since intrinsic noise in the broad representation of each individual number averages out.

The *analytic-component* of the model assumes that procedural operations (such as multicolumn addition and division, or heuristic approximations of those operations) are serially performed on symbols available in working memory^33^. As the working memory capacity is limited (about 4+/-2 items;^34^,^35^), the model predicts that as set-size increases, the distance between the subjective estimation and the true average increases, reflecting the lower relative number of samples used (Fig. 2 Lower Panel, right-hand; red line; see *Computational Model Section* for additional description). Furthermore, the model assumes that, at each sequence, the quantity of items available in working memory is subject to some trial-to-trial variability (e.g.,^36^), predicting a positive correlation between accuracy and RT, as they both increase with the number of samples that the system has used in the estimation.

The combined model, which integrates both strategies to account for the performance of the participants, assumes that a set-size-dependent parameter determines which of the two strategies is utilized in each trial (exclusive dual-process). This model provides a quantitative account of the data, which is presented after the experimental results.

## Experimental results

In Exp. 1 (skewed) and Exp. 2 (normal) the presentation rate was 500 ms per item. Our main interest is the dependency of the accuracy of the estimates (see quantification of accuracy below) and their RT on set-size. As there was no difference in the dependency of these DVs on set-size, in the two experiments [F(2, 34) = 0.95; p = 0.4 of the interaction of experiment-type with set size on accuracy; F(2, 34) = 0.84; p = 0.44 of the interaction of experiment-type with set size on RT], the analysis is reported collapsed across the two (other than when mentioned explicitly).

### Sensitivity to Numerical Averages

The participants exhibited above-chance sensitivity to the arithmetic averages of the presented number sequences, as evident by using two different measures that are computed on a subject by subject manner: (1) the correlation across trials between the sequence’s average and participant’s evaluations was high (chance involves a null-hypothesis according to which the participant’s response in a given trial is uncorrelated with the specific values presented in that trial, but may reflect the general statistics across the experiment, thus predicting zero correlation) [Pearson correlation = 0.66; SD = 0.11; p < 0.0001; for all participants; left panel in Fig. 3 shows the performance of a single participant]; and (2) for each participant, his or her square deviations between the subjective estimation and the actual average were significantly lower than simulated square deviations generated by randomly shuffling participant’s responses across trials [Actual = 61.4; Shuffled = 158.61; p < 0.01, for all participants]. This remarkable sensitivity to the numerical average of the sequence was also found for the 16-number trials, separately: (1) participants’ correlation was significant [r = 0.64 (SD = 0.12); p < 0.05, for all participants]; and (2) square deviations were smaller as compared to shuffled responses [Actual_16 = 58.1; Shuffled_16 = 139.64; p < 0.05, for all participants, except one, who nevertheless was not discarded from analysis]. In order to exclude the possibility that participants were merely picking a random number from the sequence, we compared square deviations between low-range and high-range sequences (median split of sequence range, defined as the difference between the maximal and minimal numerical values of the sequence). We found no difference in square deviations between low and high range sequences [Low = 60.66; High = 63.11; t(35) = −0.8; p = 0.43], suggesting that participants did not choose a random number from within the sequence.

To test whether participants systematically under- or overestimate the means, we have computed each participant’s average signed deviation from the actual means. An unbiased observer should exhibit no such deviation. We found a small, yet significant negative deviation [−0.79; t(35) = −2.37; p = 0.023, as compared to 0], suggesting that participants underestimate the sequences’ average. Since in Exp. 1 we used sequences with a skewed distribution of numerical values (see Method), we were also able to distinguish between an estimation that is based on arithmetic average as opposed to the median of the sequence, despite the fact that no trial-by-trial feedback, based on the actual averages, was delivered (see Method). We compared the square of the deviations of each participant’s evaluations from the sequence’s mean and median and found that the former was smaller than the latter [Mean = 59.58; Median = 98.4; p < 0.01 for all 18 participants]. We show in the Suppl., that participants’ estimations are based on both digits, rather than only on the decimal digit of each number.

Taken together, these results suggest that participants are sensitive to the statistical average of multiple rapidly presented two-digit numbers.

### Set-Size Effects

We quantify participants’ accuracy by taking the square-root of the mean square deviations (hereafter RMSD) of the subjective estimations from the sequences’ averages: 
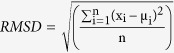
; where for each trial, 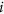, 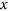 is the subjective estimation and 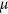 is the sequence’s arithmetic mean (note that higher values of RMSD imply lower accuracy). We found that accuracy changed as a function of set-size [repeated measure ANOVA with the within subject factor of set-size; F(2, 70) = 6.22; p = 0.003], indicating a non-monotonic dependency (see Fig. 3): RMSD in set-size 8 (M_8 = 8.1) was higher compared with both set-size 4 [M_4 = 7.35; t(35) = 3.48; p = 0.001; as compared to set-size 8] and set-size 16 [M_16 = 7.5; t(35) = 4.42; p =0.004; as compared to set-size 8]. Moreover, accuracy in the 16-number condition did not differ from accuracy in 4-number condition [p > 0.45], although analytically averaging 16 numbers is more difficult and would lead to a larger accumulated error and slower RT as compared to 4 numbers (contradictory to results; see next section).

This non-monotonic dependency is inconsistent with either the predictions of the intuitive or the analytical system operating alone, but is consistent with a dual-process account of numerical averaging (see *Computational Model Section*; and black line in Fig. 3, right panel). According to this account, participants rely on analytic/sequential thinking in set-size 4 and on the intuitive/holistic estimations in set-sizes 8 and 16.

Importantly, this non-monotonic effect is not the result of averaging two monotonic linear effects as this pattern is seen at the individual participant level (most participants – ~70% – exhibit this non-monotonic function of accuracy/set-size). Additional support for the hypothesis that participants employed an analytic strategy in the 4-number condition is obtained from the observation that the proportion of trials in which participants’ evaluations were perfect (deviation = 0) was significantly higher in the 4-number condition, relative to the 16-number condition [Perfect_4 = 0.15; Perfect_16 = 0.11; t(35) = −2.37; p = 0.023; error rates were arcsine transformed prior to statistical analysis].

### Response-times

As the non-monotonic set-size-accuracy relation implies that participants rely on different mechanisms when evaluating the average of 4 and 16 numbers, we further hypothesized that participants’ response times (RTs) would also differ between the set-size conditions, as the intuitive system is more rapid than the analytic system^22^,^23^. Indeed, we found that RTs decrease monotonically [repeated measure ANOVA with the within subject factor of set-size; F(2, 70) = 32.86; p < 0.0001; see Fig. 4]. Post-hoc comparisons reveal that in the 4-number condition RTs were much slower than those observed in the 8-number condition [RT_4 = 6.63 s; RT_8 = 4.78 s; t(35) = 5.36; p < 0.0001] as well as in the 16-number condition [RT_16 = 4.38 s; t(35) = 6.1; p < 0.0001] and that RTs in the 8-number condition were also significantly slower than in the 16-number condition [t(35) = 4.54; p < 0.0001].

Based on this dual account model, we can make a prediction for the interaction between RT and accuracy in the task. While the intuitive component predicts RT-indifference to set-size, the analytic component predicts that the longer the RT the more accurate the response (longer RTs reflect a larger number of samples held in working-memory; we assume variability across trials in this capacity). To test this we have compared RT-RMSD correlation measures: as predicted, only in the 4-number condition was the correlation between RT and RMSD significantly negative [r = −0.07; t(35) = −3.16; p = 0.003; as compared to 0]. As a complement measure, we have compared accuracy between slow and fast trials (RT median split, per each set-size) under each set-size condition and found significant RT-accuracy interaction [ANOVA; F(2, 70) = 4.93; p = 0.01; Fig. 4]. Post-hoc comparisons reveal significant improvement with higher RT in the 4-number condition [4_Fast = 7.64; 4_Slow = 6.94; t(35) = −2.35; p = 0.025; see Fig. 4]. No such effect was found in the 8- and 16-number conditions [8_Fast = 8.33; 8_Slow = 7.69; t(35) = 1.85; p = 0.07;16_Fast = 7.31; 16_Slow = 7.62; t(35) = −1.34; p = 0.19].

Moreover, we found that participants who were slower to answer the set-size 4 problems, as quantified by their ratio of mean-RT in the 4 and the 16 conditions, were more accurate in the 4-condition [Pearson correlation between RT_4/RT_16 and RMSD = −0.54; p < 0.001; see Figure S1 in Suppl.; no significant correlations were found in the 8- and 16-number conditions]. This suggests that the participants who “took more time” in the 4-number condition, did so in order to perform more operations, resulting in better accuracy in that condition.

### Temporal Bias

If indeed participants rely on analytic calculations of the sequences’ average in the 4-number condition, we should observe a biased (non-flat) temporal weighting profile of the presented numbers. This is due to the fact that analytic (explicit) processes rely on content available in working memory, which is usually recency-biased^37^,^38^. We ran separate regressions for each of the set-size conditions, using the sequence’s numbers as predictors. While we found a significant recency bias in all set-size conditions [ANOVA of within subject unstandardized regression coefficients; 4-numbers F(3, 105) = 7.43; p = 0.0001; 8-numbers F(7, 245) = 8.37; p < 0.0001; 16-numbers F(15, 525) = 1.86; p = 0.0025; see Fig. 5] the magnitude of the recency was highest at set-size 4, and lowest at set-size 16; see Fig. 5). Moreover, we found that individual recency bias (see Suppl. for description of the methodology used to quantify this measure) negatively correlates with accuracy (positively with RMSD) in the 4–number condition (and to a lesser extent in the 8-number condition), but not in the 16-number condition [Pearson correlations: 4 = 0.61; p = 0.0001; 8 = 0.34; p = 0.045; 16 = 0.2; p = 0.22]. This suggests that the recency observed in 4-number condition is most likely to stem from trials in which only a small subset of (mostly) recent items were maintained in WM distorting the evaluation, while the smaller recency observed in the 16-number condition reflects the general temporal decay profile of the neurons’ ensemble activity.

Furthermore, we found that the analytic/intuitive RT ratio (RT-4/RT-16), correlates negatively with the individual recency bias [Pearson correlation = −0.33; p = 0.049; no significant correlations were found in the 8- and 16-number conditions]. This indicates that participants who were slower in the 4-number condition based their estimation on most of the values shown, and thus suffered less from recency in that condition. Thus, it is likely that the RT ratio described above reflect individual differences in the amount of items held in working memory.

Taken together, these results demonstrate that participants are able to spontaneously tap onto the appropriate system in the two extreme conditions: when the information-load is grossly within the limits of working memory capacity (i.e., the 4-number condition) participants rely mostly on analytic operations to calculate the numerical average, as evident by slow RTs, positive RT-accuracy correlation and a deteriorative recency bias. Conversely, when the amount of information clearly overflows the analytic capacities (i.e., the 16-number condition) participants mostly rely on intuitive processes, as evident in their fast RTs, RT-accuracy invariability and relatively unbiased temporal weighting that does not impair accuracy.

In Exp. 1 and 2 we set no exogenous limitations on the participants’ processing time, an advantage which afforded the employment of analytic thinking in set-size 4. In experiments 3 (N = 18) and 4 (N = 18; see Method section), we tested whether by accelerating the processing time, either by presenting the numbers at an extremely rapid rate (Exp. 3; 100 ms per numeral), or by setting a stringent RT limitation (Exp. 4; 2.5 sec), would shift the mechanism to the intuitive mode^39^, at all set sizes. This should lead to a monotonically increasing accuracy as a function of set-size. In addition we wanted to probe the ability to make average estimations of even more rapid numerical sequences (Exp. 3).

### Experiments 3 and 4

Even when presentation time is extremely fast (Exp. 3), or response deadline is stringent (Exp. 4), the participants exhibited a remarkable, above-chance, sensitivity to the averages of the number sequences: (1) the correlation across trials between the sequences’ actual average and the participant’s evaluations is significant [Pearson correlation; Exp. 3: M_Correlation = 0.44; SD = 0.17; p < 0.05; for all participants, except one; Exp.4: M_Correlation = 0.49; SD = 0.14; p < 0.005; for all participants]; and (2) for each participant, his or her square deviations between subjective estimation and the actual average are significantly lower than simulated deviations generated by randomly shuffling participant’s responses across trials [Exp. 3: Actual = 90; Shuffled = 147; p < 0.05, for all participants, except three; we discard one participant from further analyses for being at chance performance in both measures. Discarding this data did not influence the significance of the statistical results reported below; Exp. 4: Actual = 94; Shuffled = 166; p < 0.05, for all participants]. We found no difference in square deviations between low and high range sequences [Exp. 3; Low_Range = 87.98; High_Range = 88.92; t(16) = −0.17; p = 0.87; Exp. 4; Low_Range = 98.25; High_Range = 89.82; t(17) = 1.51; p = 0.15], suggesting that participants did not pick a random number from within the sequence.

To test whether participants systematically under- or overestimate the means, we have computed each participant’s average signed deviation from the actual means. We found a negative deviation suggesting a systematic underestimation [Exp. 3; −2.17; t(16) = −2.69; p = 0.02; Exp. 4; −2.22; t(17) = −3.45; p=0.003; t-tests as compared to 0].

### Set-Size Effects

In contrast with Exp. 1 and 2, and as predicted by a single component account, based on population average, accuracy increased monotonically with set-size in Exp. 3 and 4 [repeated measure ANOVA with the within subject factor of set-size; Exp. 3: F(2, 32) = 12.74; p < 0.0001; Exp. 4: F(2, 34) = 14.85; p < 0.0001; see Fig. 6]: accuracy in set-size 4 was lower compared with set-size 8 and set-size 16 [Exp. 3: RMSD_4 = 10; RMSD_8 = 9.3; RMSD_16=8.5; Exp. 4: RMSD_4 = 10.47; RMSD_8 = 9.32; RMSD_16 = 8.5]. In addition, unlike Exp. 1 and 2, the fraction of trials in which evaluations were perfect in set-size 4 was lower than in set-size 16, in Exp.3 [Perfect_4 = 0.07789; Perfect_16 = 0.1; t(16) = −2.53; p = 0.02] and not different in Exp. 4 [Perfect_4 = 0.089; Perfect_16 = 0.085; t(17) = 0.45; p = 0.65; error rates were arcsine transformed prior to statistical analysis].

### Response-times

As the monotonic set-size-accuracy relation implies that participants rely on a single intuitive component when response-time is limited, we further hypothesized that participants’ RTs would differ little between the set-size conditions. While a small speedup with set-size was found (indicating the lack of a speed-accuracy tradeoff), the RT differences were much smaller compared with Exp. 1 and 2, especially, in the set-size 4 condition (see cyan and red lines in Fig. 4, left panel). Also, unlike in Exp. 1 and 2, within subject RT-RMSD correlations were null, for all set-size conditions and there was no interaction between speed and accuracy in the different set-size conditions [ANOVA of the RT*RMSD interaction between set-sizes; Exp. 3: F(2, 32) = 0.24; p = 0.79; Exp. 4; F(2, 34) = 0.19; p = 0.83]. These results suggest that the increased presentation rate or response time deadline shifted participants towards intuitive averaging in all conditions.

### Accounting for the Data Using a Two-Component Computational Model

To account for the results of Exp. 1-4, we have developed a computational model which consists of 2 independent components – an intuitive averaging component based on population coding and an analytic component which employs serial arithmetic operations on numbers available in working memory. In addition, motor-noise is a free parameter common to both components.

### The Intuitive Population Coding component

For the intuitive numerical averaging process, we adapted a population vector model^32^. Each number (10-90), defines a distinct Gaussian distribution (SD/width *w*) over the neural network. Upon the presentation of a number, each unit/neuron responds probabilistically, by triggering a number of spikes that is sampled from a Poisson distribution with a mean, λ, determined by the corresponding numerical tuning-curve (see Fig. 2B). Each successive number presented triggers an additional, accumulated probabilistic neural activation. At the display’s offset, a unit sums each neuron’s firing-rate multiplied by its preferred number and divided by the sum of the overall network’s activity (see eq. 1). The product is the neuron representing the activation weighted average. Finally, this neuron’s preferred number (Gaussian’s tuning curve peak) is perceived as the sequence’s average.

#### Equation 1:


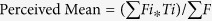


[where for each neuron 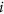, 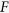: firing rate;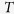: preferred number].

### The analytic component

In the analytic component of the model, estimations are the result of ideal calculations made on items available in working memory or on heuristic approximations of them (for example, a ‘rough arithmetic’ heuristic, in which each number is rounded. This introduces an additional noise parameter per operation. The model simulation is carried out with a zero value of this parameter, but the results are very similar with small heuristic-based noise). We assume that on each trial the working memory capacity is determined by sampling a value from a Gaussian distribution with the average of 4^34^,^35^ and SD as a free parameter. The sampled value is rounded to a positive integer to represent a discrete item (see Fig. 2, left lower panel).

We fitted the two-component model to the data observed in Exp. 1-4 (see Suppl., for fitting procedure and results) and found that the model is able to account for the non-monotonic relationship between accuracy (RMSD) and set-size observed in Exp. 1-2 (see Fig. 3, black line) as well as for the monotonic improvement in accuracy with set-size in Exp. 3-4 (see Fig. 7). The model accounts for the non-monotonic accuracy with set-size in Exp. 1-2 as a result of its changing strategy: analytic for set-size 4 and intuitive for set-sizes 8 and 16. On the other hand, in Exp. 3-4, which involves strict response deadline, the model relies solely on the intuitive component, which predicts a monotonic improvement with set-size.

## Discussion

We found that human participants have a remarkable ability to estimate the average of rapid sequences of two-digit numbers, at presentation rates that stretch from 2 to 10 items/sec. Importantly, at moderate presentation rates (2/sec), the relation between set-size and accuracy is not monotonic: accuracy decreases from set-size 4 to set-size 8, yet increases from set-size 8 to set-size 16 (Fig. 3). This pattern is predicted by a dual-, but not a single-component account of numerical averaging, which is based on the distinction drawn between approximate/intuitive and exact/analytic numerical cognition^19^. Under this scheme intuitive averaging is the result of perceptual-like population-based averaging, while analytic averaging relies on serial symbolic-based operations or heuristics, mediated by working memory. As we showed in our simulation study (Fig. 2 and *Computational Model Section*), the two components predict opposite effects of set-size on accuracy, allowing the dual system to account for the observed non-monotonic pattern. In particular, analytic processes become less accurate with set size (due to the WM-capacity, they work with a lower fraction of the total values that need to be averaged), while the intuitive system, modeled as a population-coding of analogous quantity/numerosity, gains precision with increasing set size, as uncorrelated noise at the single-item level averages-out.

According to our dual component model, participants are able to adaptively select the strategy (analytical vs. intuitive) that fits to the task contingency and demands (set-size, presentation-rate or response deadline). In particular, they carry out analytical calculation with a small set-size and at a moderate presentation rate (2/sec), but they spontaneously switch to intuitive computations at a high set size or high presentation rate (10/sec). Additional support for this interpretation is given by the estimation response time (RT). First, RTs in the 4-number condition were much slower than in the 16-number condition. Second, only in the 4-number condition there was a significant positive correlation between RT and accuracy (more operations undertaken improve the estimation). As a consequence of this adaptive strategy, participants are able to enhance overall performance, as compared to reliance either on intuition or analytic thinking alone. Third, we found that, when experimental limitations were set either on presentation time (Exp. 3) or on response time (Exp. 4), participants exhibit a monotonic improvement in accuracy as set-size increases (and little set-size difference in RTs; see Fig. 4 cyan line). This result is predicted by a single intuitive component and is in agreement with studies showing that the extraction of statistical properties, such as instance-frequency or the average size/diameter of circles is more efficient (i.e., faster and more accurate) in larger set-sizes^40^,^41^,^42^.

These results provide critical support in favor of the influential distinction drawn between approximate and exact numerical cognition^19^, and propose an explicit computational account, motivated by neurophysiological data. This computational model extends the processes of numerical cognition to the averaging of sequences of numbers – an operation which is crucial for the formation of preferences and decision-making^1^,^2^,^3^,^4^. The model provides further behavioral predictions as for the expected RMSD at additional set-sizes: for example, it predicts that RMSD will be smaller (accuracy higher) for 2 samples as compared to 4, and that RMSD will be lower (accuracy lower) for 12 samples as compared to 16. At the neural level, the model predicts that in intuitive averaging tasks (e.g., Exp. 3-4), when presented with two extreme numerical values (e.g., 10 and 90), the most active neural representation in the parietal cortex will appear during the encoding of the sequence around the average (50) rather than at the specific values. Future studies are also needed to examine whether, as our model predicts, activation accumulates during numerical averaging, in face of known adaptation effects taking place when participants view adjacent numbers passively^43^. Future versions of the models may include a sub-additive summation of activation to account for adaptation effects.

Recent research provides complementary results supporting the distinction between intuitive/approximate and analytical/exact strategies of numerical averaging. By contrasting average-estimations under explicit instructions to rely, either on intuition, or on computation it was found that while the computational strategy is more accurate than the intuitive one, at low set-size, the situation reverses at high set-size (Rusou, Usher & Zakay, under review). The present results are consistent with this, but further suggest that reliance on each of these mechanisms is flexible and depends upon task contingencies such as the number of samples and amount of available processing time. The extent to which these spontaneous transitions extend to other domains such as decision making and perception remains to be investigated. Our paradigm may also facilitate the study of dyscalculia, which has been shown to involve impairments in both analytic and approximate numerical processing (e.g., 7). For example, it may allow to establish whether dyscalculia patients suffer from an inability to spontaneously adapt to task-contingencies and to rely on the appropriate mechanism under different conditions.

## Materials and Methods

### Participants

Overall, 72 participants participated in the four experiments (*N*_*1*_ = 18 (Mean age = 23.8; SD = 2.5); *N*_*2*_ = 18 (Mean age = 24; SD = 2.2); *N*_*3*_ = 18 (Mean age = 22.8; SD = 1.9); 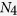
=18 (Mean age=23.1; SD=1.5); different participants in each experiment). All participants were undergraduate students recruited through the Tel Aviv University Psychology Department’s participant pool, were naive to the purpose of the experiment and had normal, or corrected-to-normal, vision. Informed consent was obtained from all subjects. Participants were awarded either course credit for their participation or a small financial compensation (40 NIS; equivalent to about $10). Participants received a performance-dependent bonus of additional 10-20 NIS. All procedures and experimental protocols were approved by the ethics committee of the Psychology department of Tel Aviv University (Application 743/12). All experiment were carried out in accordance with the approved guidelines.

### Stimulus Materials and Procedure

**Figure 1:**
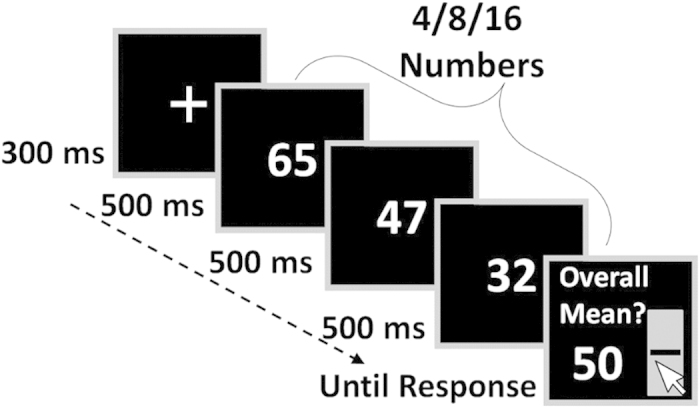
Schematic illustration of an experimental trial (Exp. 1-2). Each trial begins with a 300 ms fixation cross, after which a sequence of two-digit numbers is presented (500 ms per numeral). The sequence set-size was 4, 8 or 16 (randomly between trials). The only instructions participants received were to convey as accurately as possible the sequence’s average, by vertically sliding a mouse-controlled bar set on a number ruler between 0 and 100 (the number corresponding to the bar’s location was concurrently displayed).

**Figure 2:**
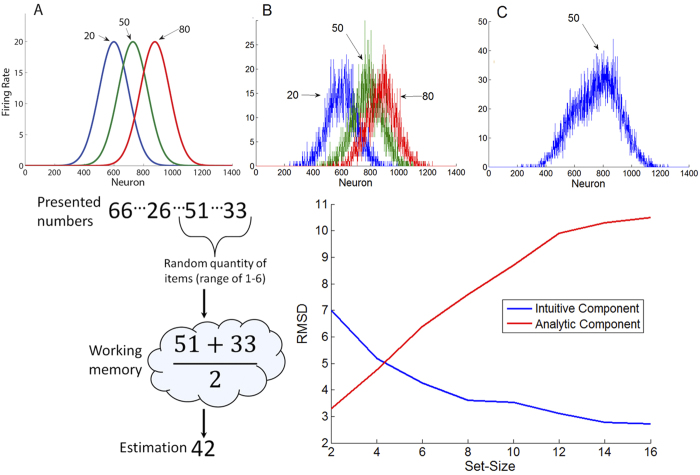
Dual-component model for numerical averaging. Upper Panel: neuronal population vector underlying intuitive numerical averaging: **A**) numerosity tuned neural detectors; **B**) Presented numbers activate noisy neural detectors (superimposed); **C**) The activation profile of the weighting units summing each neuron’s firing-rate, the center-of-mass unit represents the perceived numerical average. Lower Panel, left-hand: illustration of the analytic component: in each trial a varying subset of the presented numbers (range 1-6) reaches working memory; Lower Panel, right-hand: qualitative predictions of the two components of the averaging model: Simulated RMSD (square-root of mean square deviations) as a function of the number of items presented, for the analytic (red) and the intuitive (blue) components; tuning-curve width = 20; working-memory variability = 1; motor noise = 3; see Computational Model Section for a full description of the model).

**Figure 3:**
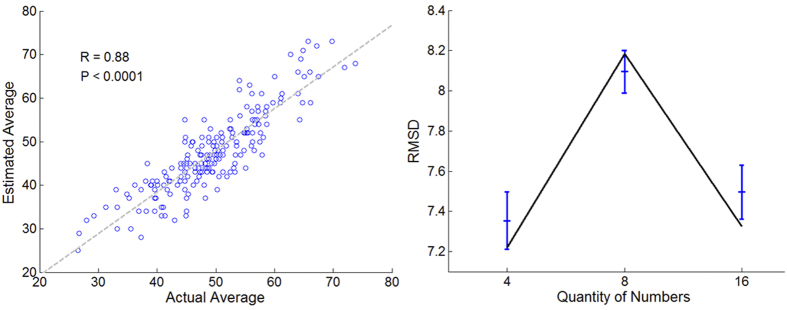
Left-Panel: Performance of a single-subject in Exp. 2. The scatter plot depicts the participant’s evaluations (y-axis) for each of the presented number-sequence averages (x-axis). Dashed line is the regression line (R = 0.88; mean R for the 36 participants = 0.66). Right-Panel: observed accuracy (RMSD) for the different set-size conditions (4, 8 and 16 numbers) in Exp. 1-2 (blue bars). Accuracy in estimating the numbers’ average deteriorates when set size increases from 4 to 8, yet improves when set size increases from 8 to 16, suggesting a non-monotonic relation between set-size and accuracy. This pattern is captured by a dual-component model (black line). Estimations of the intuitive component parameters were obtained by fitting the intuitive component to Exp. 4 (see Suppl., for a description of the fitting procedure).

**Figure 4:**
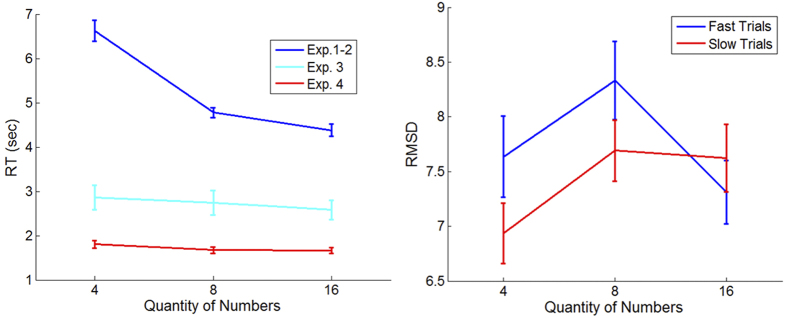
Response times (RTs) and RT-Accuracy interaction under the different set-size conditions. Left Panel: RTs strongly decrease as a function of set-size in Exp. 1-2 and minimally in Exp. 3-4; Right Panel: Faster trials (blue line; RT median split per each set-size) are less accurate in set-size 4 and 8, while being slightly more accurate in set-size 16 Exp. 1-2.

**Figure 5:**
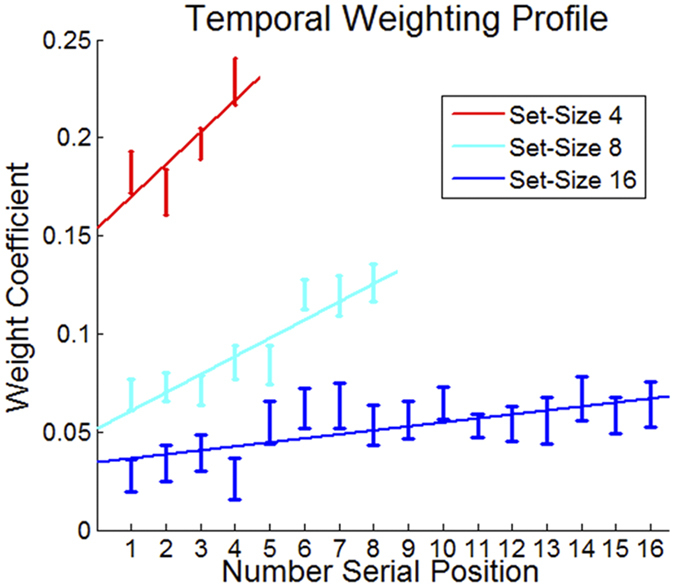
Temporal-weighting profile (unstandardized regression weights) of the numbers under the different set-size conditions. Participants’ evaluations were more influenced by the recent items in the sequence (i.e., recency bias) under the 4- and 8-number conditions, but not under the 16-number condition.

**Figure 6:**
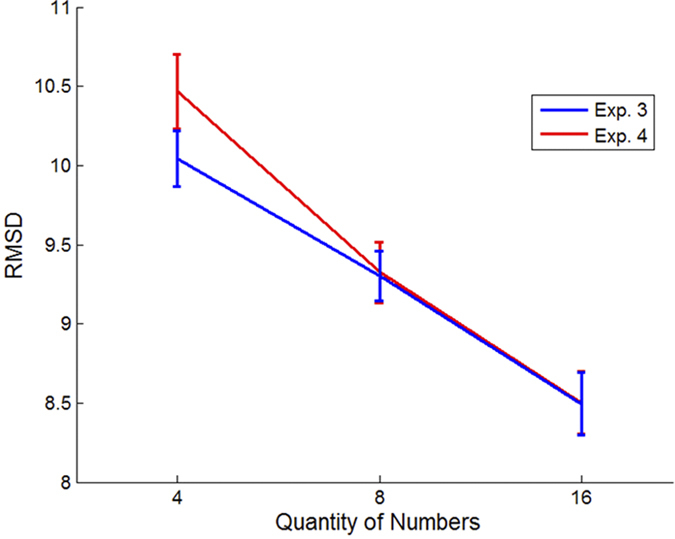
Observed accuracy (RMSD) in Exp. 3 (blue bars) and 4 (red bars), under the different set-size conditions (4, 8 and 16 numbers): When processing time is limited, accuracy in estimating the numbers’ average improves with set size, suggesting a single intuitive-component account of numerical averaging.

**Figure 7:**
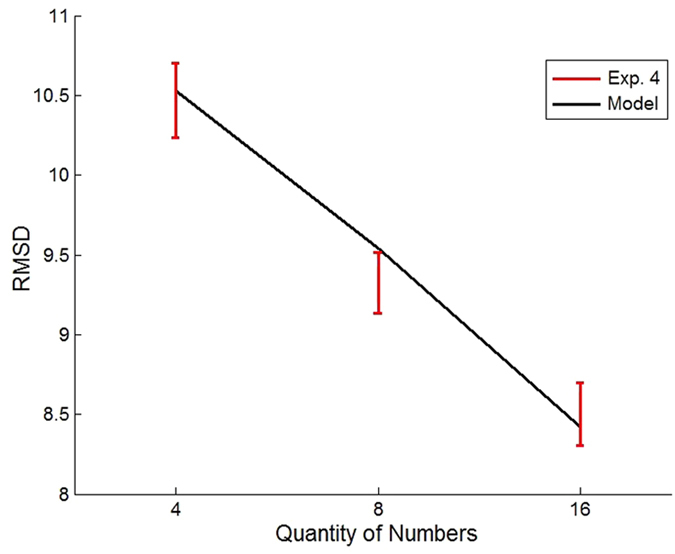
Observed and Predicted accuracy (RMSD) in Exp. 4 (red bars), under the different set-size conditions (4, 8 and 16 numbers: The monotonic pattern is captured by a one-component model of intuitive numerical averaging using population vector (black line).

The basic set-up of a trial is depicted in Fig. 1. In Exp. 1 and 2, each trial began with a central fixation cross (300 ms) after which a sequence of two-digit numbers was presented (white Arabic numerals on black background; each number displayed for 500 ms; without blank ISIs). The sequence set size (i.e., the quantity of displayed numbers) was 4, 8 or 16 - randomly between trials. The only instructions participants received were to convey as accurately as possible the sequence’s average, by vertically sliding a mouse-controlled bar set on a number ruler between 0 and 100 (the number corresponding to the bar’s location was concurrently displayed) and pressing the left mouse button when reaching the desired number. In Exp. 1 and 2, we explained to the participants that their only objective is to be as accurate as possible and offered payoff for accuracy. After completing 20 practice trials, participants underwent 120 experimental trials divided into 6 blocks. Each block terminated with performance-feedback (block-average correlation) and a short, self-paced break. To generate each sequence of numbers in Exp. 1, 3 and 4 we predefined four triangular skewed-density distributions, ranged between 10 and 90; with means of: 40, 46, 54, or 60. Each sequence was sampled from one of the four distributions (random between trials). In case two identical numbers were sampled successively, the entire sequence was shuffled in order to prevent successive presentation. In Exp. 2, we used normal (Gaussian) underlying distributions to generate each sequence of numbers (means of the distributions were randomly sampled between 35 and 65; SD of distributions was 30). All stimuli were generated using Matlab© and were presented on a gamma-corrected ViewSonic (Walnut, CA) 17-in. monitor viewed at a distance of 41 cm. The screen resolution was set to 1,024 × 768 pixels, and the monitor had a refresh rate of 60 Hz.

### Data and Statistical Analysis

We obtained participants’ evaluation and response time (RT; measured from sequence’s offset until mouse button press) in each trial. All regression weights used in the different analyses or depicted in figures are unstandardized beta coefficients. We discarded data from one participant in Exp. 3 for being at chance performance on both chance-level measures (i.e., correlation and shuffled responses); no other data was discarded in all experiments.

## Additional Information

**How to cite this article**: Brezis, N. *et al.* Adaptive Spontaneous Transitions between Two Mechanisms of Numerical Averaging. *Sci. Rep.* **5**, 10415; doi: 10.1038/srep10415 (2015).

## Supplementary Material

Supplementary Information

## References

[b1] Bechara A., Damasio H., Tranel D. & Damasio A. R. Deciding advantageously before knowing the advantageous strategy. Science 275, 1293–1295 (1997). 9036851 10.1126/science.275.5304.1293

[b2] Anderson N. H. Application of an additive model to impression formation. Science 138, 817–818 (1962). 17820996 10.1126/science.138.3542.817

[b3] Hertwig R. & Erev I. The description-experience gap in risky choice. Trends in cognitive sciences 13, 517–523, 10.1016/j.tics.2009.09.004 (2009). 19836292

[b4] Tsetsos K., Chater N. & Usher M. Salience driven value integration explains decision biases and preference reversal. Proceedings of the National Academy of Sciences of the United States of America 109, 9659–9664, 10.1073/pnas.1119569109 (2012). 22635271 PMC3386128

[b5] Levin I. P. Averaging processes and intuitive statistical judgments. Organizational Behavior and Human Performance 12, 83–91 (1974).

[b6] Malmi R. A. & Samson D. J. Intuitive averaging of categorized numerical stimuli. Journal of Verbal Learning and Verbal Behavior 22, 547–559 (1983).

[b7] Stanescu-Cosson R. *et al.* Understanding dissociations in dyscalculia A brain imaging study of the impact of number size on the cerebral networks for exact and approximate calculation. Brain 123, 2240–2255 (2000). 11050024 10.1093/brain/123.11.2240

[b8] Hyde D. C. & Spelke E. S. Neural signatures of number processing in human infants: evidence for two core systems underlying numerical cognition. Developmental science 14, 360–371, 10.1111/j.1467-7687.2010.00987.x (2011). 21399717 PMC3050652

[b9] Feigenson L., Dehaene S. & Spelke E. Core systems of number. Trends in cognitive sciences 8, 307–314, 10.1016/j.tics.2004.05.002 (2004). 15242690

[b10] Pica P., Lemer C., Izard V. & Dehaene S. Exact and approximate arithmetic in an Amazonian indigene group. Science 306, 499–503 (2004). 15486303 10.1126/science.1102085

[b11] Dehaene S., Molko N., Cohen L. & Wilson A. J. Arithmetic and the brain. Current opinion in neurobiology 14, 218–224 (2004). 15082328 10.1016/j.conb.2004.03.008

[b12] Wood G. *et al.* All for one but not one for all: How multiple number representations are recruited in one numerical task. Brain research 1187, 154–166 (2008). 18022606 10.1016/j.brainres.2007.09.094

[b13] Klein E., Nuerk H.-C., Wood G., Knops A. & Willmes K. The exact vs. approximate distinction in numerical cognition may not be exact, but only approximate: How different processes work together in multi-digit addition. Brain and cognition 69, 369–381 (2009). 18929439 10.1016/j.bandc.2008.08.031

[b14] Ariely D. Seeing sets: Representation by statistical properties. Psychological science 12, 157–162 (2001). 11340926 10.1111/1467-9280.00327

[b15] Chong S. C. & Treisman A. Representation of statistical properties. Vision research 43, 393–404 (2003). 12535996 10.1016/s0042-6989(02)00596-5

[b16] Piazza M., Izard V., Pinel P., Le Bihan D. & Dehaene S. Tuning curves for approximate numerosity in the human intraparietal sulcus. Neuron 44, 547–555 (2004). 15504333 10.1016/j.neuron.2004.10.014

[b17] Gigerenzer G. & Selten R. Bounded rationality: The adaptive toolbox . (Mit Press, 2002).

[b18] Rosenberg-Lee M., Lovett M. C. & Anderson J. R. Neural correlates of arithmetic calculation strategies. Cognitive, Affective, & Behavioral Neuroscience 9, 270–285 (2009). 10.3758/CABN.9.3.270 19679763

[b19] Dehaene S. The number sense: How the mind creates mathematics . (Oxford University Press, 2011).

[b20] Betsch T. & Glöckner A. Intuition in judgment and decision making: Extensive thinking without effort. Psychological Inquiry 21, 279–294 (2010).

[b21] Evans J. S. B. & Stanovich K. E. Dual-process theories of higher cognition advancing the debate. Perspectives on Psychological Science 8, 223–241 (2013). 10.1177/1745691612460685 26172965

[b22] Kahneman D. Thinking, fast and slow . (Macmillan, 2011).

[b23] Evans J. S. B. Dual-processing accounts of reasoning, judgment, and social cognition. Annu. Rev. Psychol. 59, 255–278 (2008). 18154502 10.1146/annurev.psych.59.103006.093629

[b24] Nieder A. & Miller E. K. A parieto-frontal network for visual numerical information in the monkey. Proceedings of the National Academy of Sciences of the United States of America 101, 7457–7462, 10.1073/pnas.0402239101 (2004). 15123797 PMC409940

[b25] Nieder A. & Miller E. K. Coding of cognitive magnitude: compressed scaling of numerical information in the primate prefrontal cortex. Neuron 37, 149–157 (2003). 12526780 10.1016/s0896-6273(02)01144-3

[b26] Piazza M., Izard V., Pinel P., Le Bihan D. & Dehaene S. Tuning curves for approximate numerosity in the human intraparietal sulcus. Neuron 44, 547–555, 10.1016/j.neuron.2004.10.014 (2004). 15504333

[b27] Harvey B., Klein B., Petridou N. & Dumoulin S. Topographic representation of numerosity in the human parietal cortex. Science 341, 1123–1126 (2013). 24009396 10.1126/science.1239052

[b28] Dotan D., Friedmann N. & Dehaene S. Breaking down number syntax: Spared comprehension of multi-digit numbers in a patient with impaired digit-to-word conversion. Cortex 59, 62–73 (2014). 25133926 10.1016/j.cortex.2014.07.005

[b29] Verguts T. & Fias W. Representation of number in animals and humans: a neural model. Journal of Cognitive Neuroscience 16, 1493–1504 (2004). 15601514 10.1162/0898929042568497

[b30] Van Opstal F., de Lange F. P. & Dehaene S. Rapid parallel semantic processing of numbers without awareness. Cognition 120, 136–147 (2011). 21489415 10.1016/j.cognition.2011.03.005

[b31] Dehaene S. Subtracting pigeons: logarithmic or linear? Psychological science 12, 244–246; discussion 247 (2001). 11437308 10.1111/1467-9280.00343

[b32] Georgopoulos A. P., Schwartz A. B. & Kettner R. E. Neuronal population coding of movement direction. Science 233, 1416–1419 (1986). 3749885 10.1126/science.3749885

[b33] Logie R. H., Gilhooly K. J. & Wynn V. Counting on working memory in arithmetic problem solving. Memory & cognition 22, 395–410 (1994). 7934946 10.3758/bf03200866

[b34] Luck S. J. & Vogel E. K. The capacity of visual working memory for features and conjunctions. Nature 390, 279–281 (1997). 9384378 10.1038/36846

[b35] Cowan N. Metatheory of storage capacity limits. Behavioral and brain sciences 24, 154–176 (2001). 10.1017/s0140525x01003922 11515286

[b36] Shafi M. *et al.* Variability in neuronal activity in primate cortex during working memory tasks. Neuroscience 146, 1082–1108 (2007). 17418956 10.1016/j.neuroscience.2006.12.072

[b37] Atkinson R. C. & Shiffrin R. M. Human memory: A proposed system and its control processes. Psychology of learning and motivation 2, 89–195 (1968).

[b38] Davelaar E. J., Goshen-Gottstein Y., Ashkenazi A., Haarmann H. J. & Usher M. The demise of short-term memory revisited: empirical and computational investigations of recency effects. Psychological Review 112, 3 (2005). 15631586 10.1037/0033-295X.112.1.3

[b39] Rand D. G., Greene J. D. & Nowak M. A. Spontaneous giving and calculated greed. Nature 489, 427–430 (2012). 22996558 10.1038/nature11467

[b40] Pitz G. F. The sequential judgment of proportion. Psychonomic Science 4, 397–398 (1966).

[b41] Erlick D. E. Absolute judgments of discrete quantities randomly distributed over time. Journal of Experimental Psychology 67, 475 (1964). 14171240 10.1037/h0042698

[b42] Robitaille N. & Harris I. M. When more is less: Extraction of summary statistics benefits from larger sets. Journal of vision 11, 18 (2011). 22031908 10.1167/11.12.18

[b43] Piazza M., Pinel P., Le Bihan D. & Dehaene S. A magnitude code common to numerosities and number symbols in human intraparietal cortex. Neuron 53, 293–305 (2007). 17224409 10.1016/j.neuron.2006.11.022

