# Supplementary material for: Adaptive Spontaneous Transitions between Two Mechanisms of Numerical Averaging
Source: Sci Rep. 2015 Jun 4;5:10415. doi: 10.1038/srep10415 (PMC4455229; doi:10.1038/srep10415)
Supplement: Supplementary Information [file srep10415-s1.pdf]

Adaptive Spontaneous Transitions between Two Mechanisms of Numerical Averaging

Noam Brezis[1]*, Zohar Z. Bronfman[1, 2]* & Marius Usher[1, 3]

Supplementary Materials

Sensitivity to numerical averages

To explore the possibility that participants processed only the decimal digit, we compared the mean square deviations of each participant from the sequences' actual mean and from the sequences' decimal digits mean and found that the former was significantly smaller than the latter [M_Sqr_Dev_Full=74.77; M_Sqr_Dev_Dec=61.4; t(35)=4.4; p<0.0001]. In addition, we compared for each set-size two competing linear regression models of the observed evaluations: a regression in which the sequence numbers are predictors and a regression in which only the decimal digits of the sequence numbers are predictors. In order to compare between the models, we used logit-transformation of the R^2. We found that the transformed R^2 of the full model was higher than that of the decimal-only model [M_R^2_4_Full=0.55 M_R^2_4_Dec=0.5; t(35)=5.52; p<0.0001; M_R^2_8_Full=0.44 M_R^2_8_Dec=0.37; t(35)=5.87; p<0.0001; M_R^2_16_Full=0.56 M_R^2_16_Dec=0.53; t(35)=4.42; p<0.0001].

Individual recency bias

In order to quantify each participant's temporal bias for each set-size condition (i.e., to estimate his or her primacy or recency), we computed the ratio between the average of the first half and second half of the non-standardized regression coefficients, per set-size, given by:

$$\frac{\sum_{\left(\frac{n}{2}\right)+1}^{n} \beta_i}{\sum_{1}^{\left(\frac{n}{2}\right)} \beta_i}$$

RTs ratio and accuracy

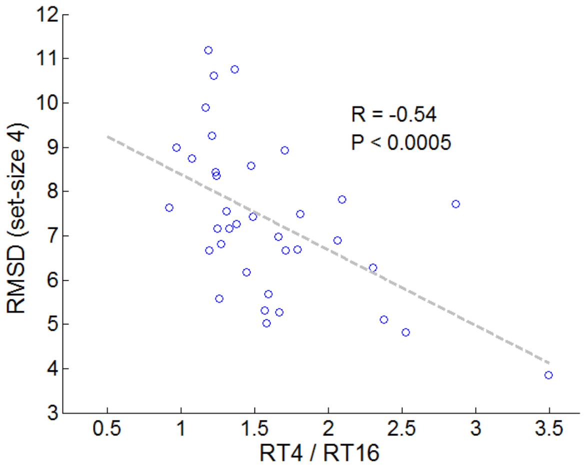

Figure S1 – correlation between subjects' relative speed at set-size 4 (as compared to RT in set-size 16) and accuracy (RMSD) at set-size 4 (Exp. 1-2; N=36)

Fitting Procedure (Figure 3)

We obtained an estimate of the tuning curve width parameter of the intuitive component (*w*) by fitting this component to observed evaluations in the 3 set-size conditions in Exp. 4, using the maximal likelihood method. We found that *w*=41 and motor noise = 7.8 (log_likelihood=-14,140; BIC=28,297). Next we fitted the intuitive component, with this value of *w* to the 16-number condition of Exp. 1-2 and extracted the motor noise (noise=6.35; log_likelihood=-7,210; BIC=14,436). With these parameters, the intuitive component predicts that RMSD_16=7.31 and RMSD_8=8.18 (see Figure-3 main text). We next fitted RMSD in the 4-number condition with the analytic component (using the same motor noise as obtained in the 16-number condition). We found that SD of working memory span within trials=0.74 and RMSD_4=7.22 (log_likelihood=-7,058; BIC=14,123).
